# Microwave-Enhanced Photolysis of Norfloxacin: Kinetics, Matrix Effects, and Degradation Pathways

**DOI:** 10.3390/ijerph14121564

**Published:** 2017-12-14

**Authors:** Wenchao Liao, Virender K. Sharma, Su Xu, Qingsong Li, Lei Wang

**Affiliations:** 1 School of Environmental Science and Engineering, Xiamen University of Technology, Xiamen 361024, China; xusu@xmut.edu.cn (S.X.); liqs@xmut.edu.cn (Q.L.); wangl@xmut.edu.cn (L.W.); 2 Program for the Environment and Sustainability, Department of Environmental and Occupational Health, School of Public Health, Texas A&M University, College Station, TX 77843, USA; vsharma@sph.tamhsc.edu

**Keywords:** antibiotics, photolysis, oxidation, microwave, synergistic effect

## Abstract

Degradation of norfloxacin (NOR) was studied using a combination of microwave and UV irradiation methods (MW/UV process). Remarkable synergistic effect was found between MW and UV light. The removal rate with the MW/UV process was much faster than that with UV light irradiation only. Degradation of NOR followed second-order kinetics and ~72% of NOR could be removed in the first 5 min of MW/UV reaction. Influence of inorganic ions (cations (K^+^, Mg^2+^, Ca^2+^, Cu^2+^) and anions (Cl^−^, SO_4_^2−^, NO_3_^−^, CO_3_^2−^)), humic acid (HA) and surfactants (cation, anion, and non-ionic) on the degradation of NOR by the MW/UV process was investigated. Among the ions, Cu^2+^ and NO_3_^−^ ions inhibited the degradation of NOR. The presence of HA and surfactants in water showed a slight inhibition on the NOR removal. Furthermore, the NOR degradation in the MW/UV process was primarily caused by the ·OH-photosensitization steps. Seven intermediates formed by the oxidation of NOR were identified and three reaction pathways were proposed. Removals of NOR in tap water (TW), synthetic wastewater (WW), river water (RW), and seawater (SW) were also studied, which demonstrated that the MW/UV process was an effective oxidation technology for degrading fluoroquinolone antibiotics in different water matrices.

## 1. Introduction

Antibiotics have received increasing concern as emerging pollutants in recent years for their ubiquitous occurrence and ecological risks [1,2]. Among them, fluoroquinolones (FQs) are some of the most widely used broad-spectrum antibiotics for curing bacterial infections in humans, livestock and aquaculture [3,4]. Fluoroquinolones, especially norfloxacin (NOR), have been detected at relatively high levels in various water matrices such as wastewater effluents [5,6,7], surface water [8,9] and even drinking water [10]. Continuous release of FQs into the aquatic environment might trigger the development of antibiotic resistant bacteria and genes [11,12,13,14,15], which would be even bigger threats to human health and ecosystem safety. Most FQs cannot be effectively removed by microbial processes in conventional wastewater treatment plants, due to the stability and poor biodegradability of the quinolone ring [16,17]. It is thus of utmost importance to seek highly efficient technologies to remove FQs in water [18]. The focus of the present paper was on applying photolysis to degrade a selected fluoroquinolone in water.

Many FQs exhibit different degrees of photosensitive property and can be potentially degraded by direct photolysis or photosensitization [19,20,21]. Various techniques have been combined with photolysis to improve the quantum yield in photodegradation, which include photocatalysts, electricity, ultrasound and the addition of oxidants (e.g., H_2_O_2_ and ozone) [22,23,24,25]. The current paper explored the combination of microwave irradiation with UV photolysis, named the MW/UV process, to degrade antibiotics present in different water matrices.

Microwave (MW)-enhanced TiO_2_ photocatalysis using MW-discharged electrodeless lamps (MDELs) as the light source has attracted increasing attention for improving the degradation efficiency of various organic pollutants in aqueous and atmospheric environments [26,27,28,29,30,31]. Studies have demonstrated that the irradiation of MW on TiO_2_ could form hot spots in the photocatalysts, which could increase the surface defects on TiO_2_ and improve the production of additional charge carriers and trap sites to enhance the photocatalytic degradation rate of pollutant [32,33]. However, microwave-enhanced photocatalytic processes are still not practical for large-scale wastewater treatment plants because of the high cost, inconvenient recycle and potential toxicity of the existing inorganic photocatalysts. Meanwhile, MW-promoted photolysis method (or MW/UV process) without the use of any catalysts and additives represented an alternate oxidation technology to degrade recalcitrant organic pollutants. Examples included dyes [34,35], 2,4-dichlorophenoxyacetic acid [36,37], 4-chlorophenol [38], butyl acetate [39], and H_2_S [40]. For economy and efficiency sake, the MW/UV process was a more competitive and promising technique for the treatment of unbiodegradable organic pollutants in large-scale wastewater treatment plants. However, the studies to date are not sufficient. The present study was the first example of the application of the MW/UV process to degrade FQ antibiotics in various water matrices. The mechanism of the microwave and MDELs discharged UV light coupled process was also demonstrated.

Aims of the current study were: (i) degradation of NOR by the MW only, UV only, and MW/UV processes to demonstrate synergistic effect between MW and MDELs UV light; (ii) varying the MW power to optimize the conditions to efficiently degrade NOR in water; (iii) investigating the effect of ions (K^+^, Mg^2+^, Ca^2+^, Cu^2+^, Cl^−^, SO_4_^2−^, NO_3_^−^, CO_3_^2−^), HA, and surfactants (cationic, anionic, and non-ionic) to learn the influence of wastewater components on the degradation efficiency of NOR by the MW/UV process; (iv) seek the degradation of NOR present in tap water (TW), wastewater (WW), river water (RW) and seawater (SW) to learn the applicability of the MW/UV process to remove the antibiotics from polluted water, and (v) propose reaction pathways for tthe degradation of NOR in the MW/UV process by identifying the intermediates of the reactions.

## 2. Materials and Methods

### 2.1. Chemicals

All the chemicals of analytical grade and solvents of chromatographic grade were obtained from different commercial suppliers and were used without further purification. Specifically, norfloxacin (NOR, 98%) and humic acid (>90%) were purchased from Aladdin Industrial Corporation (Shanghai, China). Methanol was acquired from Tedia Company Inc. (Cincinnati, OH, USA). Other chemicals (KCl, MgCl_2_, CaCl_2_, CuCl_2_, NaCl, NaSO_4_, NaNO_3_, Na_2_CO_3_, cetyltrimethyl ammonium bromide (CTAB), sodium dodecyl sulfonate (SDS), Tween 60, NaN_3_) were obtained from Sinopharm Chemical Reagent Co. Ltd. (Shanghai, China).

Generally, NOR solution was prepared in deionized water (DW, Milli-Q, Billerica, MA, USA). Inorganic salts, humic acid, and surfactants, were added into the NOR solution to study matrices effects on the degradation of NOR by MW/UV process. The concentrations of the additives were illustrated in Section 3.2. Tap water (TW) was obtained from the tap in the chemical lab of Xiamen University of Technology (Xiamen, China). Synthetic wastewater (WW) was prepared in the lab by adding 10 mg (NH_4_)_2_SO_4_, 10 mg KH_2_PO_4_, 20 mg CO(NH_2_)_2_ and 480 mg C_6_H_12_O_6_ into 1 L deionized water and stirring until all solids dissolved. River water (RW) was sampled from the north part of the Xinglinwan Reservoir (Xiamen, China), and seawater SW) was taken from Tong’an Bay in Xiamen (Xiamen, China). The chemical compositions of the five water matrices are given in Table 1.

### 2.2. Degradation of NOR in the MW/UV Process

The experimental setup of the MW/UV process consisted in an atmospheric microwave system (APEX, Preekem Scientific Instruments Co. Ltd., Shanghai, China) and two U-style MDELs (Shanghai Jiguang Special Illumination Instruments Factory, Shanghai, China). The MDELs filled with mercury (60 mg per lamp) and argon (1 Torr per lamp) had maximum wavelength at 254 nm. The intensity of the UV light of the MDELs linearly increased with the microwave power (Equation (1)):

(1) $$y=0.0912x-12.18\;R^{2}=0.9873$$

where *y* represents the UV light intensity of two MDELs, mW/cm^2^ and *x* represents the microwave power, W.

During the experimental procedures, two MDELs were dipped into the NOR solution in a cylindrical glass reactor (diameter 250 mm, height 200 mm), and the whole reactor was placed into the center of the APEX microwave system. When microwave was turned on, the MDELs was lighten, and the NOR solution could receive simultaneous irradiation of microwave and UV light. The microwave power applied ranged from 300 W to 600 W, and the UV light intensity was enhanced using Equation (1). In most cases, circulated degradation experiments were carried out with 2 L NOR solution (5 mg/L) at a flow rate 140 mL/min. The pH of NOR solution was 6.72 and no further adjustment was made. All experiments were conducted in triplicate, and each sample was immediately subjected to analysis after collection.

In the MW only process, the APEX microwave system remained as the microwave sources (microwave power = 500 W), but no MDELs were applied. In the UV only process, the MDELs were installed onto an external microwave magnetron setup (Lvqing Industrial Microwave Equipment Factory, Shanghai, China), and the MW power in the UV process was also set as 500 W to match with the power used in the MW/UV and MW processes. In this setup, MW was not applied to the NOR solution, but only used for the excitation of the MDELs. Therefore, the effect of microwave on the NOR solution could be excluded in the UV process.

### 2.3. Analytical Procedures

Concentration of NOR was determined by high performance liquid chromatography (HPLC) pereformed on a Shimadzu LC-15C instrument (Shimadzu, Kyoto, Japan), coupled with a diode array detector. The mobile phase was 0.1% formic acid (A)/pure methanol (B) (A/B = 50/50, *v*/*v*). The column was a reversed phase Shimadzu InertSustain C18 analytical column (4.6 mm × 150 mm, particle size 5 μm) and worked at 40 °C. The flow rate of mobile phase was 1.0 mL/min. The detection of NOR was carried out at 270 nm. The analysis of total organic carbon (TOC) of NOR solution was performed using a Shimadzu TOC-V analyzer.

For identification of intermediates, the samples were withdrawn at different intervals and subjected to analysis by an Agilent 1260 HPLC (Agilent Technologies Co. Ltd., Palo Alto, CA, USA) coupled with an AB Sciex Triple Quad 3500 mass spectrometer (AB Sciex LLC., Redwood City, CA, USA). The mass spectrometer was equipped with an electrospray ionization source and functioned in positive mode. An XTerraMS C18 column (100 mm × 2.1 mm, particle size 3.5 μm, Waters Technologies Co. Ltd., Shanghai, China) was used to separate the intermediate products. The mobile phase was 0.1% formic acid (A)/pure acetonitrile (B) in a gradient elution mode. The gradient began with 100% A to 100% B in 30 min, and the mobile phase flow rate was set as 0.2 mL/min. The mass operating condition of mass spectrometer were provided in Table 2.

## 3. Results and Discussion

### 3.1. Kinetics

**Figure 1:**
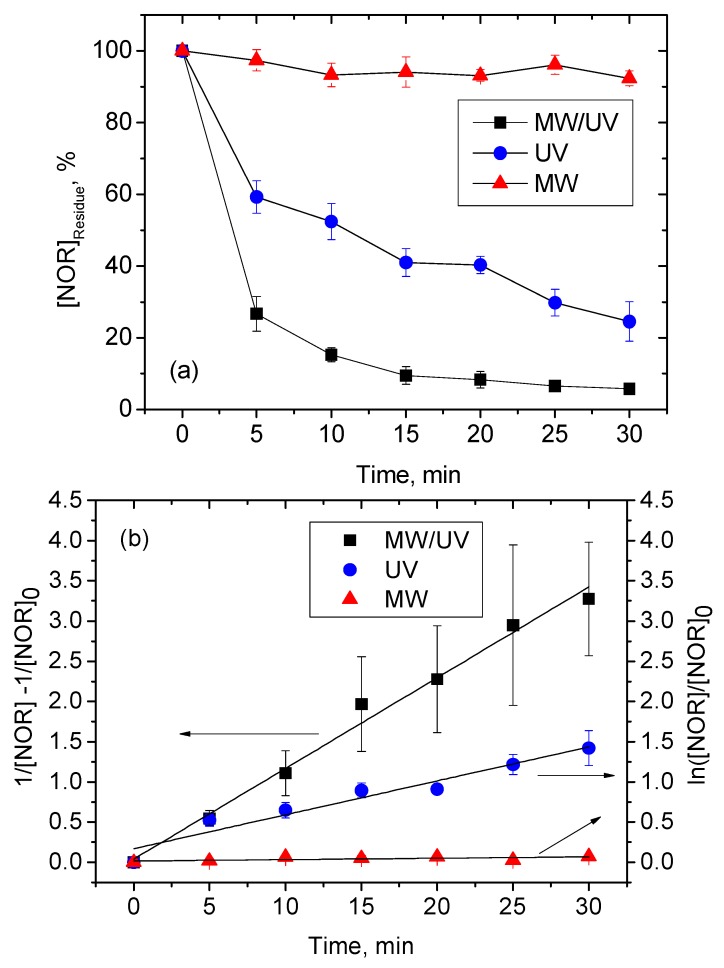
(**a**) Degradation of NOR with time by different processes and (**b**) kinetics analysis of the results obtained in (**a**). (Experimental conditions: microwave power = 500 W, UV light intensity = 31.8 mW/cm^2^, maximum wavelength = 254 nm, [NOR] = 5 mg/L, and pH = 6.72).

In initial experiments, degradation of NOR in water was sought by the MW, UV, and MW/UV processes (Figure 1a). The MW process caused little removal of NOR. Comparatively, both the UV and MW/UV processes could degrade NOR in water (Figure 1a). The MW/UV process had much higher degradation rate than the UV process (Figure 1a). 

Significantly, the degradation rate of NOR by the MW/UV process was greater than the sum of the degradation rates of the UV and MW processes. Meanwhile, the data in Figure 1a were also verified to have significant differences through single variable significance analysis (Table 3). This suggested that a combination of UV and MW processes resulted in a synergistic effect to degrade NOR in water.

The kinetics of NOR degradation by the MW, UV and MW/UV processes were analyzed by fitting the results of Figure 1a with first-order and second-order kinetic laws. The rates of the MW and UV process could be fitted by first-order kinetics while the MW/UV processes followed second-order kinetics (Figure 1b). Microwave irradiation alone had little effect on the degradation of NOR, but it could significantly accelerate the photolysis of NOR when combined with UV light irradiation. Values of the obtained rate constants were 4.2 × 10^−2^ min^−1^ and 1.1 × 10^−1^ mg^−1^ min^−1^ for the UV and MW/UV processes, respectively. The calculated values of half-lives (t_1/2_) for 5 mg/L NOR were determined as 16.4 min and 1.8 min, respectively (Table 4). Both results showed that coupled microwave and UV light irradiation could clearly improve the NOR degradation rate and shorten the reaction time. The introduction of microwave made the UV photolysis process more efficient in degrading NOR in water.

As for the reason, the lower photon energy of MW (0.0016 eV) was not able to break the covalent bonds of NOR [41]. The synergistic effect between MW and UV light irradiations might be ascribed to the directional arrangement effect of MW irradiation on polar molecules. This effect could stabilize the transition states of polar reactions and decrease the activation energies during the photolysis of NOR molecules [42]. Moreover, the high frequency MW irradiation could improve the formation of reactive oxygen species in aqueous solution [43]. Therefore, the coupling of MW irradiation with the photolysis process could achieve much higher and faster removal of NOR than traditional photolysis.

**Figure 2:**
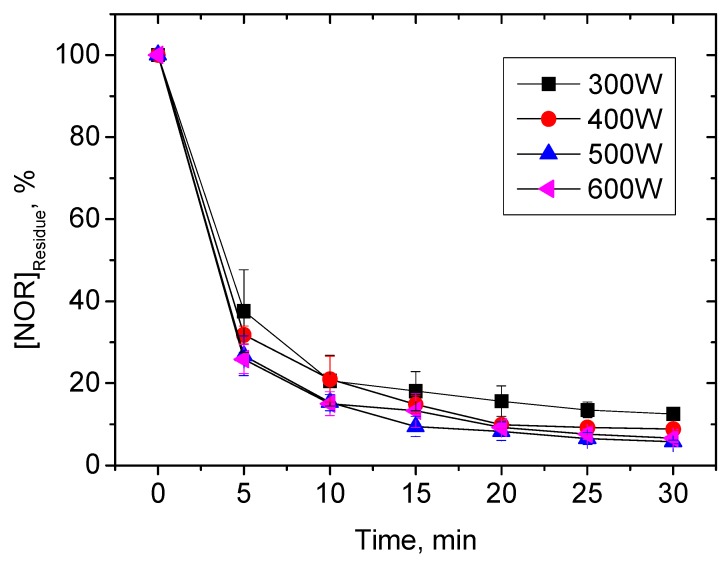
Effect of microwave power on the degradation of NOR by the MW/UV process. (Experimental conditions: microwave power/UV light intensity—300 W/17.1 mW/cm^2^, 400 W/23.0 mW/cm^2^, 500 W/31.8 mW/cm^2^, 600 W/42.0 mW/cm^2^), maximum wavelength = 254 nm, [NOR] = 5 mg/L, and pH = 6.72).

Microwaves are the only energy source in the MW/UV process. Enhancing the MW power also increased linearly the UV light intensity of the MDELs (see Equation (1)). This dual role of the MW power was an important factor in the MW/UV process. So the effect of MW power on the degradation of NOR was firstly investigated (Figure 2). Results in Figure 2 illustrated that the degradation of NOR increased with the increase of MW power. This might be attributed to the increase in the MDELs UV light intensity with the increase of the MW power. The non-thermal effect of microwave irradiation, such as decreasing activation energies of NOR degradation and increasing amounts of reactive oxygen species generation in the system, might also contribute to the enhanced NOR degradation. Significantly, when MW power was increased from 500 W to 600 W, degradation rates were similar. A 500 W MW power was optimum to remove NOR in water by the MW/UV process.

### 3.2. Effects of Different Water Constituents on NOR Degradation

#### 3.2.1. Inorganic Ions

**Figure 3:**
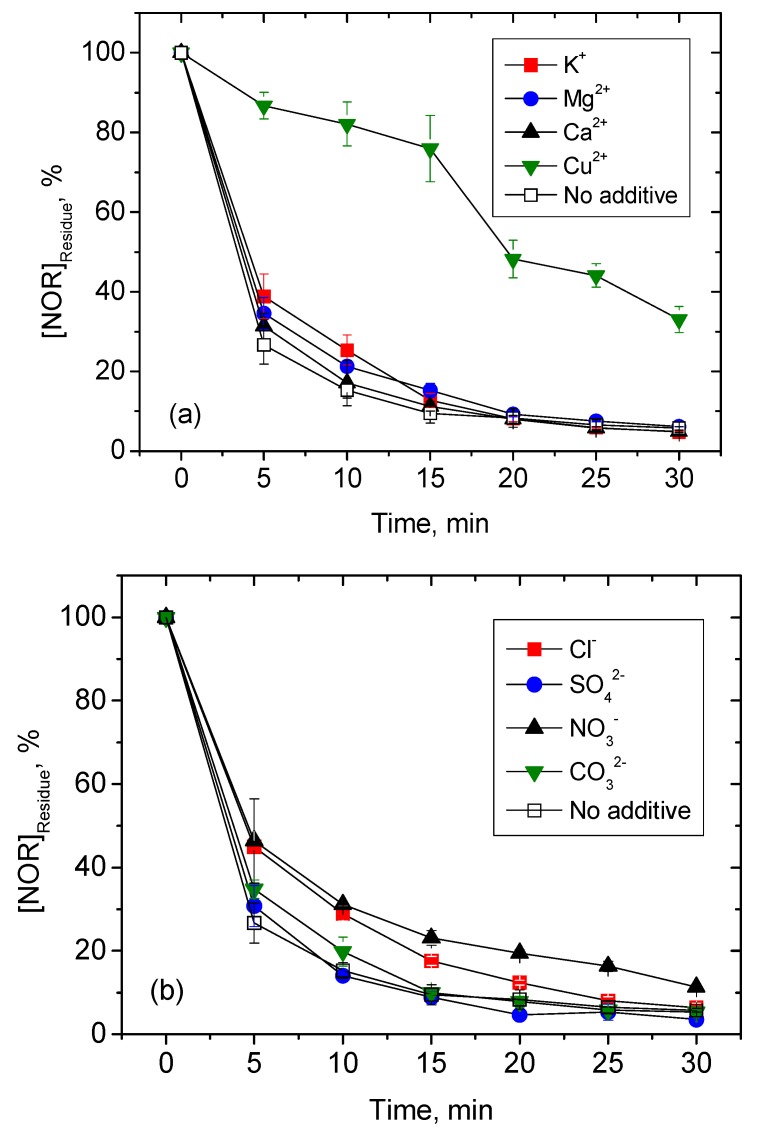
Effects of cations (**a**) and anions (**b**) on the degradation of NOR by the MW/UV process. (Experimental conditions: microwave power = 500 W, UV light intensity = 31.8 mW/cm^2^, maximum wavelength = 254 nm, [NOR] = 5 mg/L, [cation] = 5 mmol/L, and [anion] = 5 mmol/L).

Four common (monovalent and divalent) cations found in natural water matrices were added into the NOR solution to evaluate the effects of cations on degradation of NOR by the MW/UV process (Figure 3a). Most of the studied cations, except Cu^2+^, had almost no effect on the NOR removal. However, the addition of Cu^2+^ caused an average of 45% decrease on NOR degradation in the 30 min MW/UV reaction. 

The degradation trend was also quite different from the NOR degradation without cations. Transition metal ion Cu^2+^ was reported to be more likely to form complexes with fluoroquinolones [44], which might inhibit the degradation of NOR by the MW/UV process. 

The influence of four typical anions on the degradation of NOR was also studied (Figure 3b). The anions SO_4_^2−^, CO_3_^2−^, and Cl^−^ had no apparent effect on the NOR degradation. However, NO_3_^−^ showed an average of 10% decrease on the NOR removal during the 30 min MW/UV irradiation. The possible reason might be attributed to the competing light absorption between NOR and NO_3_^−^, which caused the UV light screening effect on NOR, and finally hindered the MW/UV degradation efficiency. This effect of NO_3_^−^ has also been observed in another study [45].

#### 3.2.2. DOM and Surfactants

**Figure 4:**
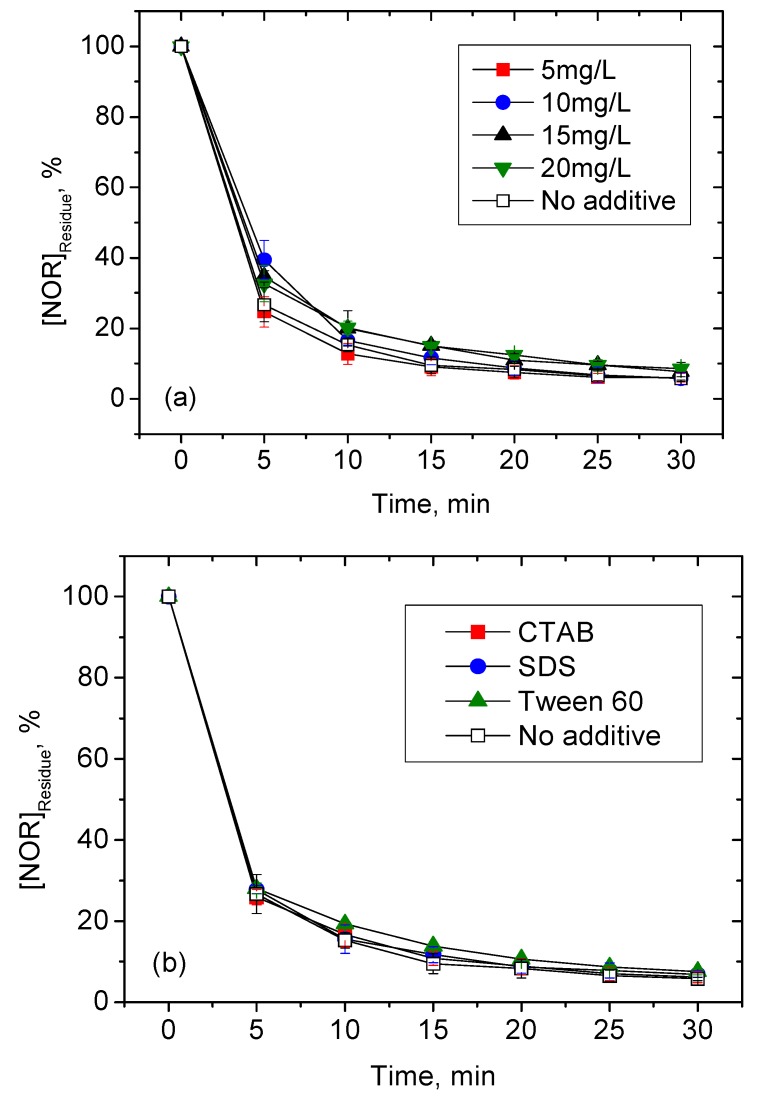
(**a**) Effect of humic acid concentration on the degradation of NOR by the MW/UV process and (**b**) Effects of different surfactants on the degradation of NOR by the MW/UV process. (Experimental conditions: microwave power = 500 W, UV light intensity = 31.8 mW/cm^2^, maximum wavelength = 254 nm, [NOR] = 5 mg/L, and [surfactant]: 5 mg/L).

As ubiquitous constituents in natural water matrices, DOM may influence the photochemical behaviors of pollutants through a variety of reactions such as photochemical sensitization, UV light screening, and quenching of reactive oxygen species (ROS) [46]. The effect of HA concentration on NOR was shown in Figure 4a. 

The overall impact of HA, ranging from 5 mg/L to 20 mg/L, on the degradation of NOR was not obvious. However, a slightly increasing inhibition trend was seen when the concentration of HA increased. It might be because the UV light screening and ROS quenching effects of HA were not significant.

Surfactants are frequently detected in natural water matrices. Effect of surfactant was also studied by adding 5 mg/L of cationic surfactant (cetyltrimethyl ammonium bromide (CTAB)), anionic surfactant (sodium dodecyl sulfonate (SDS)), and nonionic surfactant (tween 60) into NOR solution, respectively. Figure 4b demonstrates that basically all the surfactants involved played a very little inhibitory role in the MW/UV degradation of NOR. Significantly, surfactants almost had no effect on the NOR removal in water.

#### 3.2.3. Real Water Samples

**Figure 5:**
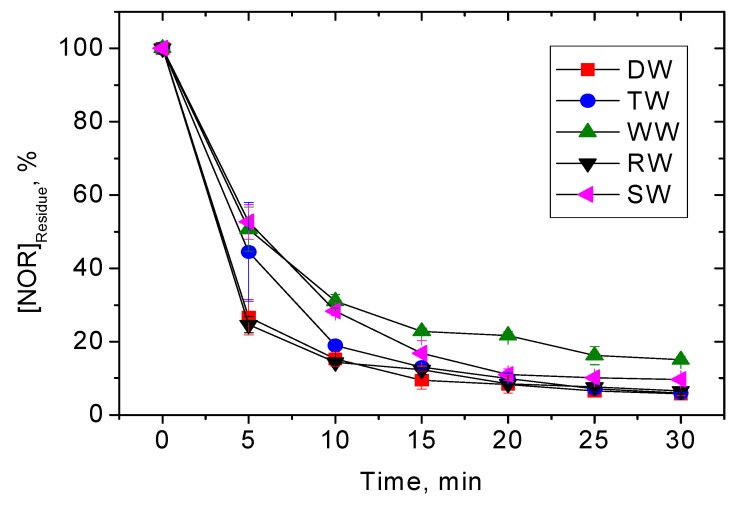
Degradation of NOR by the MW/UV process under different water matrices. (Experimental conditions: microwave power = 500 W, UV light intensity = 31.8 mW/cm^2^, maximum wavelength = 254 nm, [NOR] = 5 mg/L, DW—DI water, TW—Tap water, WW—Synthetic wastewater, RW—River water, SW—Sea water).

Finally, the feasibility of the MW/UV process on the treatment of NOR in different water matrices was examined. The results of the NOR degradation in five different kinds of water matrices DW, TW, WW, RW and SW were presented in Figure 5. High levels of NOR degradations could be obtained after 15 min of MW/UV reactions in all the examined water matrices (Figure 5). Removal of NOR followed the order as DW > RW ≈ TW > SW > WW. The best removal efficiency of NOR was in DW. NOR in more complex water matrices encountered lower degradation rate than that in DW. The NOR in WW had the lowest removal, which might be ascribed to the screening effect of NO_3_^−^ (see the highest TN value of WW in Table 1). However, although the TN value of SW was almost the same with that of RW, the NOR in SW showed lower removal than that in RW. It might be because SW had abundant of Cl^−^, which might have some inhibition on the degradation of NOR (see Figure 3b). The NOR removals in RW and TW were quite similar. The possible reason for the lower NOR removal in TW might be due to some undetected ions, like Fe^3+^ ions from the iron pipe. The Fe^3+^ ions could also easily form complex with NOR [44], and thus inhibited the degradation of NOR.

### 3.3. Reaction Pathways

Many fluoroquinolone antibiotics have been reported to undergo degradation by reactive oxygen species during self-sensitizing photolysis [47]. Experiments were performed to determine the species involved in the degradation of NOR by the MW/UV process. In this experiment, CH_3_OH and NaN_3_ were added into NOR solution, which were specific quenchers for the ·OH and ^1^O_2_ [48], respectively. 

**Figure 6:**
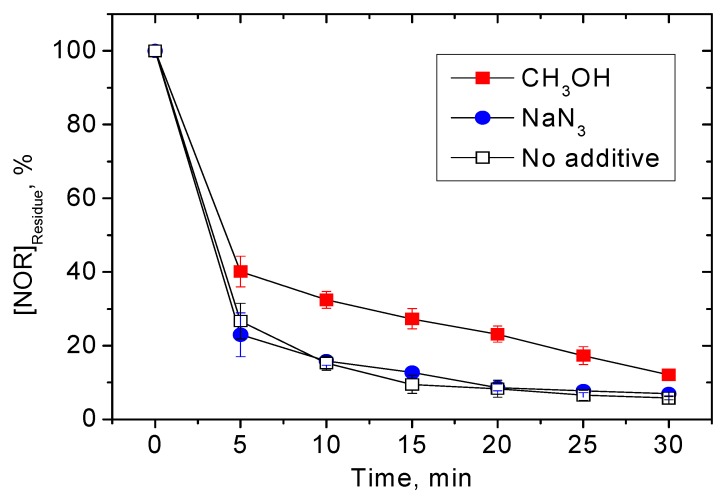
Degradation of NOR by the MW/UV process using different scavenging agents. (Experimental conditions: microwave power = 500 W, UV light intensity = 31.8 mW/cm^2^, maximum wavelength = 254 nm, [NOR] = 5 mg/L, [CH_3_OH] = 5 mg/L, [NaN_3_] = 5 mg/L).

The degradation of NOR was not affected by the addition of NaN_3_ (Figure 6), which suggested that ^1^O_2_ was not effectively participating in the MW/UV degradation of NOR. However, the addition of CH_3_OH clearly inhibited the degradation of NOR, which meant ·OH might be one of the main species causing the degradation of NOR by the MW/UV process.

**Figure 7:**
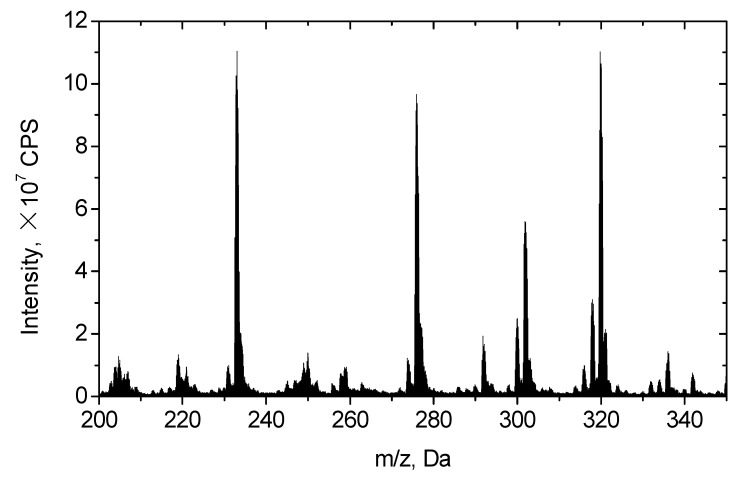
The MS spectra of NOR intermediates detected in sample withdrawn after 1 min of MW/UV reaction.

**Figure 8:**
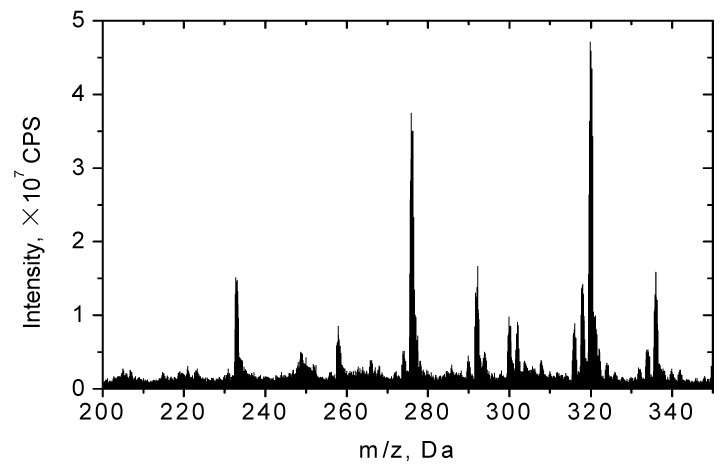
The MS spectra of NOR intermediates detected in sample withdrawn after 5 min of MW/UV reaction.

**Figure 9:**
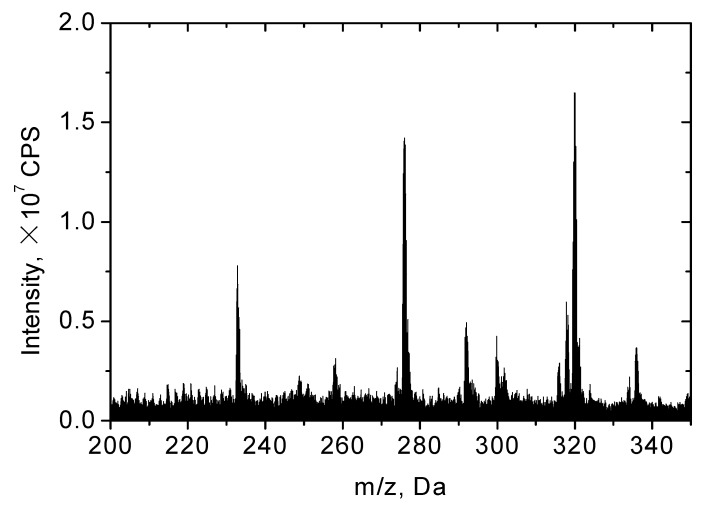
The MS spectra of NOR intermediates detected in sample withdrawn after 30 min of MW/UV reaction.

The intermediate products, formed during the MW/UV degradation of NOR in DW, were characterized by the LC-HRMS technique in a positive mode. Seven intermediates were identified, based on the corresponding molecular ions and the mass fragment ions detected by MS/MS spectra. The detailed information and proposed structure of the intermediates were summarized in Table 5 and Figure 7, Figure 8 and Figure 9.

The proposed structures of the seven intermediates were basically deduced through direct photolysis and ·OH attack on benzene ring or piperazine ring. Similar intermediates were also seen in H_2_O_2_ assisted photocatalysis [22], photoysis [21], gamma-ray irradiation [49], and electrochemical oxidation [50] of NOR.

**Scheme 1:**
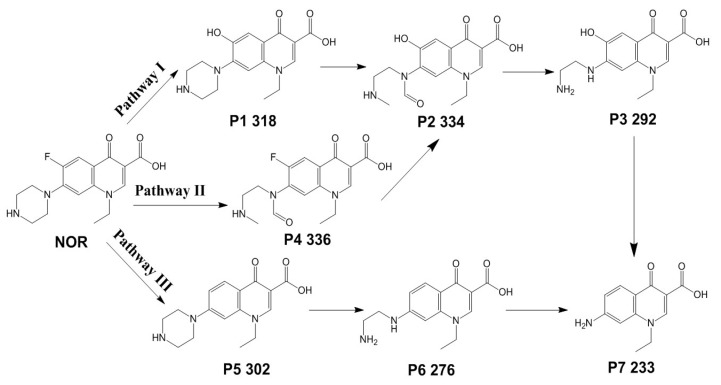
Reaction pathways of the degradation of NOR by the MW/UV process.

Three pathways for the NOR degradation in the MW/UV process are proposed based on the identified intermediates (Scheme 1). In pathway I, ·OH radicals initially attack the benzene ring of NOR, which leads to the F atom substitution with ·OH and formation of intermediate P1. This reaction has been previously reported by Kleiser et al. [51] and Chen et al. [22]. This step was followed by a ·OH attack on the piperazine ring of P1, that induces the piperazine ring opening and amide formation. Then a carbon centered radical is generated on the α position amine of the piperazine side chain, and further reacts with oxygen to produce P2 under the continuous attack of ·OH. Further attacks of ·OH on P2 subsequently form P3 and P7. 

Pathway I in this study was in consistent with the pathway I of NOR degradation in the H_2_O_2_ assisted photocatalysis process [22], but the intermediate(s) between P2 and P3, and the intermediate(s) between P3 and P7 were not detected in this study, which might be due to the fast transformation from P2 to P3, and P3 to P7 in the MW/UV process. Pathway II also initiated from the ·OH attack on NOR, but the attack position was on the piperazine ring, which resulted in product P4. The MW/UV process went through F atom substitution with ·OH and transformation of product P4 to product P2 occurred. The intermediate P3, with OH group substituting for F atom on the benzene ring of the intermediate MW 294, was clearly identified. It appeared that the defluorination might be occurring easier in the MW/UV process. This was supported by the pathway III. In Scheme 1, the C-F bond on NOR was broken under the simultaneous irradiations of microwave and UV light. The breakage of the C-F bond yielded product P5. Although the bond energy of C-F was quite high, the intense irradiation of the small amount 185 nm UV light emitted from the MDELs and the function of lower activation energy by microwave irradiation might combine to improve the defluorination of NOR [52]. P6 and P7 were then generated through the aforementioned steps in the pathway I and pathway II. The overall degradation pathways suggested that the MW/UV process was an efficient method to degrade NOR.

**Figure 10:**
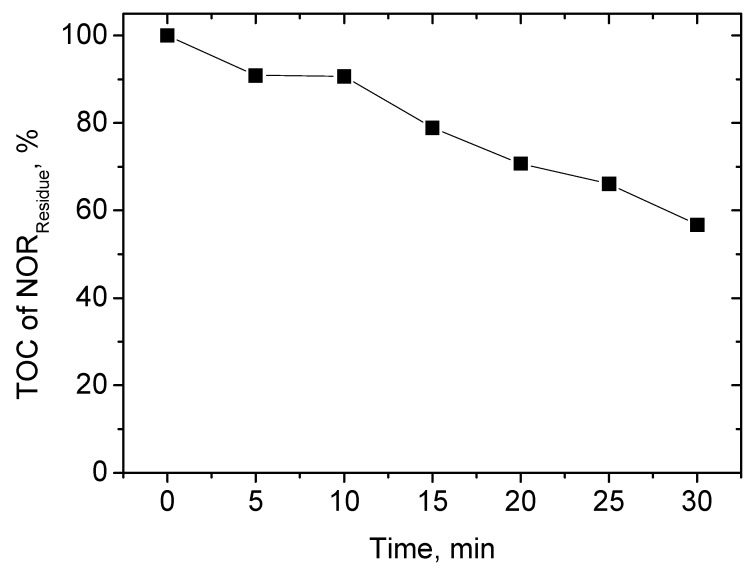
Removal of TOC during the degradation of NOR by the MW/UV process (experimental conditions: microwave power = 500 W, UV light intensity = 31.8 mW/cm^2^, [NOR] = 5 mg/L, and pH = 6.72).

Values of TOC during the degradation of NOR by MW/UV process were also determined. The levels of TOC during the 30 min of MW/UV process were shown in Figure 10. In the first 10 min, only 10% of TOC was removed from the NOR solution, followed by clearly acceleration of TOC removal in further 20 min of the MW/UV process. After 30 min of the MW/UV degradation, only 43% of the NOR TOC could be removed Overall, the results showed the gradual disintegration of NOR molecules, but 30 min of MW/UV process was not enough for the mineralization of NOR. TOC results under extended reaction time (60 min) could also be found in supplementary file.

## 4. Conclusions

A clear synergistic effect was observed in the MW/UV degradation of NOR in water. The NOR degradation followed second-order kinetics and removal of NOR at 5 mg/L could be accomplished in 15 min by the MW/UV process. Increasing the microwave power up to 500 W could increase the degradation of NOR. Among the studied inorganic ions, Cu^2+^ and NO_3_^-^ ions significantly inhibited degradation of NOR under the studied conditions. DOM and surfactants showed slight inhibition effects on the degradation of NOR by the MW/UV process. The MW/UV process was able to degrade NOR presented in real water matrices. NOR mainly underwent direct photolysis and ·OH related photosensitization in the MW/UV process. Three proposed pathways were consistent with the seven identified intermediates. The intermediates were primarily formed through defluorination and cleavage of the piperazine ring under the attack of ·OH radicals. Based on the similar core structure in FQs, the results of NOR might be extended to the degradation of other FQs in water by the MW/UV process.

## Figures and Tables

**Table 1:** Water quality indexes of different water matrices.

Parameter	DW	TW	RW	SW	WW
pH	7.2	6.7	7.6	6.6	8.0
Conductivity (μs/cm)	0.25	71.9	1627	1.07 × 10^5^	1700
Turbidity (NTU)	0.46	0.77	4.61	21.40	1.65
DO (mg/L)	87.2	82.6	77.1	80.5	1.5
TN (mg/L)	0.29	0.29	1.93	2.00	10.00
COD (mg/L)	0	47	334	1869	100

DW: deionized water; TW: tap water; RW: river water; SW: seawater; WW: synthetic wastewater; DO: dissolved oxygen; TN: total nitrogen; COD: chemical oxygen demand.

**Table 2:** MS operation conditions.

**Scan Range (m/z)**	**Drying Gas Temperature**	**Drying Gas Flow Rate**
50–350	300 °C	8 L/min
**Fragmentor**	**Nebulizer Pressure**	**Capillary Voltage**
150 V	35 psi	4000 V

**Table 3:** Significance analysis of different processes comparation data.

Source	III-Type Sum of SQUARE	df	Mean Square	F	Sig.	Partial Eta Square	Non Central Parameter	Observed Power ^b^
Calibration model	86,276.499 ^a^	20	4313.825	448.834	0.000	0.995	8976.674	1.000
Intercept	200,831.323	1	20,0831.323	20,895.577	0.000	0.998	20,895.577	1.000
V11 ^c^	22,262.405	6	3710.401	386.050	0.000	0.982	2316.301	1.000
V12 ^c^	53,814.143	2	26,907.071	2799.557	0.000	0.993	5599.115	1.000
V11*V12 ^d^	10,199.951	12	849.996	88.438	0.000	0.962	1061.258	1.000
Error	403.670	42	9.611		0.000			
Total	287,511.492	63						
Calibrated total	86,680.168	62						

^a^ R^2^ = 0.995 (Modified R^2^ = 0.993); ^b^ Calculation results by alpha = 0.05; ^c^ V11: Reaction time, V12: Different degradation processes (MW/UV, UV and MW); ^d^ V11*V12: The interaction between V11 and V12.

**Table 4:** Kinetic parameters of the NOR removals in different processes.

Process	*k*_obs_	$t_{\frac{1}{2}}$ (min)
MW/UV	(1.13 ± 0.31) × 10^−1^ mg^−1^ min^−1^	1.77 ^a^
UV	(4.22 ± 0.56) × 10^−2^ min^−1^	16.43 ^b^
MW	(1.73 ± 0.21) × 10^−3^ min^−1^	400.66 ^b^

^a^ For the MW/UV process, $t_{\frac{1}{2}}$ = $\frac{1}{c_{0}k_{\mathrm{obs}}}$, $c_{0}=5\;\mathrm{mg}/L$; ^b^ For the UV and MW processes, $t_{\frac{1}{2}}=\frac{\ln2}{k_{\mathrm{obs}}}.$

**Table 5:** The information of intermediates formed in the MW/UV degradation of NOR.

Intermediates	Molecular Ion, [M + H]^+^	Chemical Structure	Fragments (*m/z*)
NOR	320	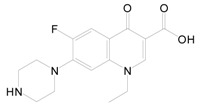	
P1	318	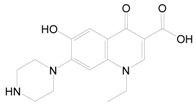	300.2, 257, 231.1
P2	334.1	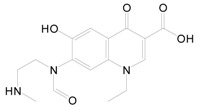	316.2, 245
P3	292	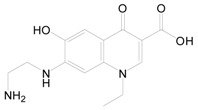	221.1
P4	336	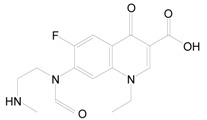	318.2, 275, 247, 233, 221.1, 205
P5	301.8	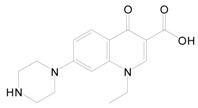	231
P6	275.9	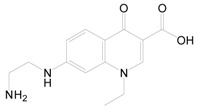	233.1, 205.1
P7	232.7	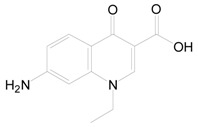	203.2, 176.1

## Supplementary Materials

Supplementary File 1

## Abbreviations

The following abbreviations are used in this manuscript:

NOR	norfloxacin
MW/UV	microwave-enhanced photolysis process
UV	Photolysis process
MW	Microwave irradiation process
MDELs	Microwave discharged electrodeless lamps
DW	Deionized water
TW	Tap water
WW	Synthetic wastewater
RW	river water
SW	Sea water
