# Supplementary material for: Microwave-Enhanced Photolysis of Norfloxacin: Kinetics, Matrix Effects, and Degradation Pathways
Source: Int J Environ Res Public Health. 2017 Dec 14;14(12):1564. doi: 10.3390/ijerph14121564 (PMC5750982; doi:10.3390/ijerph14121564)
Supplement: Supplementary File 1 [file ijerph-14-01564-s001.pdf]

International Journal of
Environmental Research and Public Health

Dark control experiments without microwave and UV light irradiation were conducted on a heating plate, and the samples were collected under the same temperatures as those in the MW/UV process. Figure SM-5 displayed the temperature increasing of NOR during the 30 min reaction in the MW/UV process. The samples in dark control experiments were withdrawn and detected when the temperatures of NOR solution were heated to the same temperatures as those under 0 min, 5min, 10min, 15 min, 20 min, 25 min and 30 min of MW/UV irradiation, respectively. The dark control results were shown in Figure SM-6. NOR seemed almost stable under conventional heating in this experiment, which demonstrated that thermal effect did little impact on the degradation of NOR.

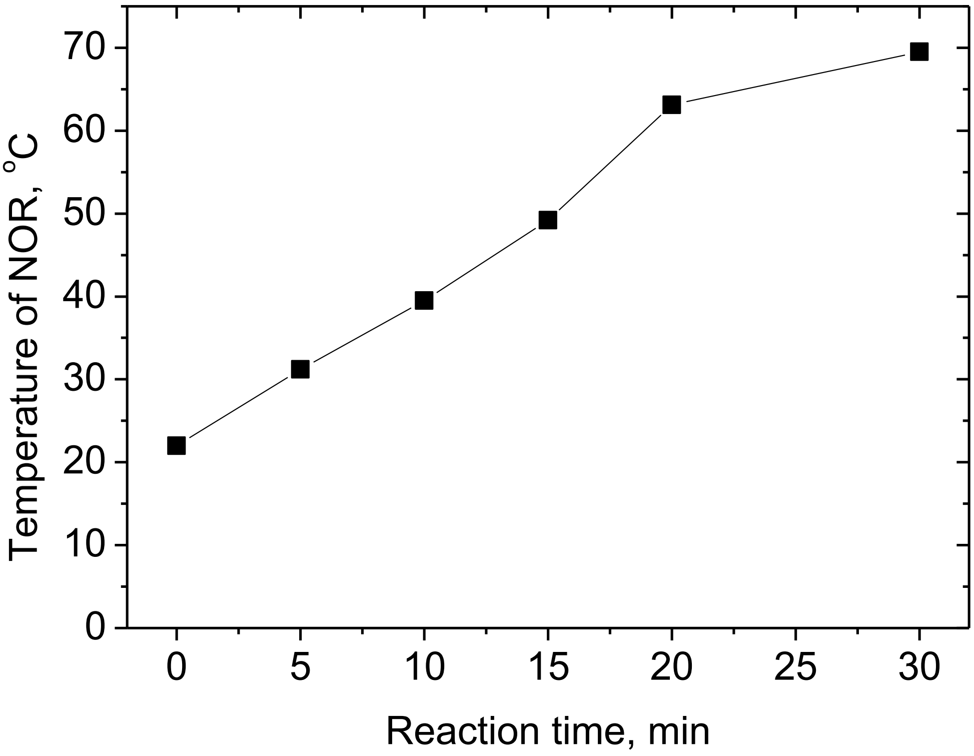

Figure SM-5. Temperature increasing of NOR in the MW/UV process. (Experimental conditions: microwave power = 500 W, UV light intensity = 31.8 mW/cm^2, maximum wavelength = 254 nm, [NOR] = 5 mg/L, and pH = 6.72).

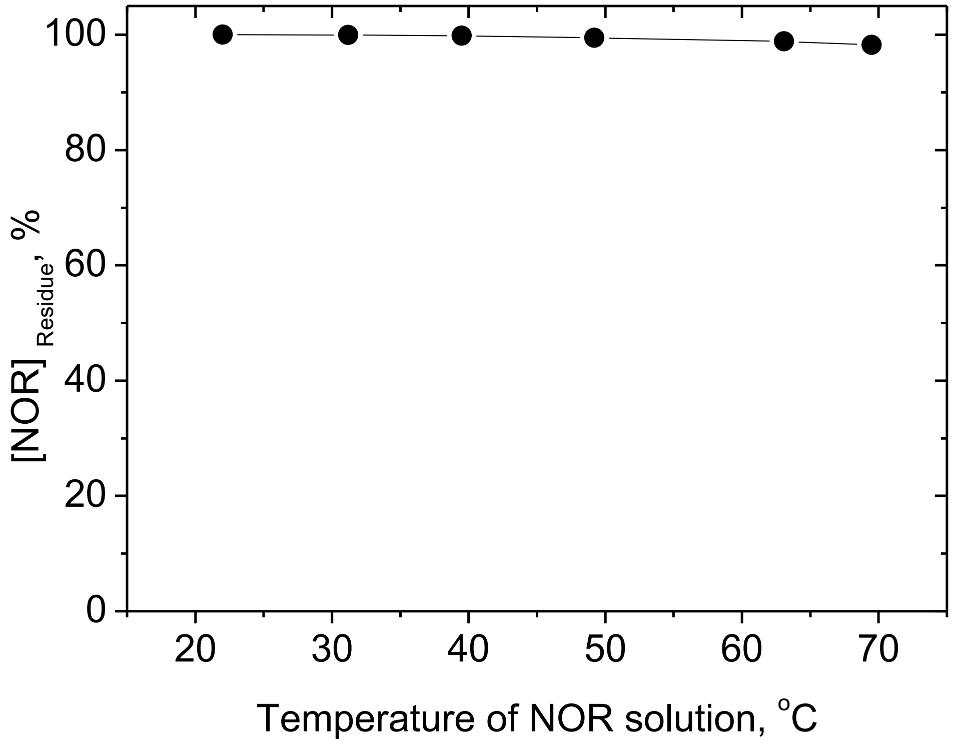

Figure SM-6. NOR degradation in conventional heating process. (Experimental conditions: heating plate power = 1500 W, [NOR] = 5 mg/L, and pH = 6.72).

The degradation of NOR by MW/UV process was extended to 60 min to investigate the further mineralization of NOR. The levels of TOC during the 60 min of MW/UV process were shown in Figure SM-7. When the reaction time was extended to 60 min, the residue of TOC could reduce to 24%. Results showed the gradual disintegration of NOR molecules in the MW/UV process. It was deduced that extending the reaction time could finally obtain complete mineralization of NOR, but using the MW/UV process as a pre-treatment and combined it with a normal microbial treatment would be more economic in practical NOR wastewater treatment project.

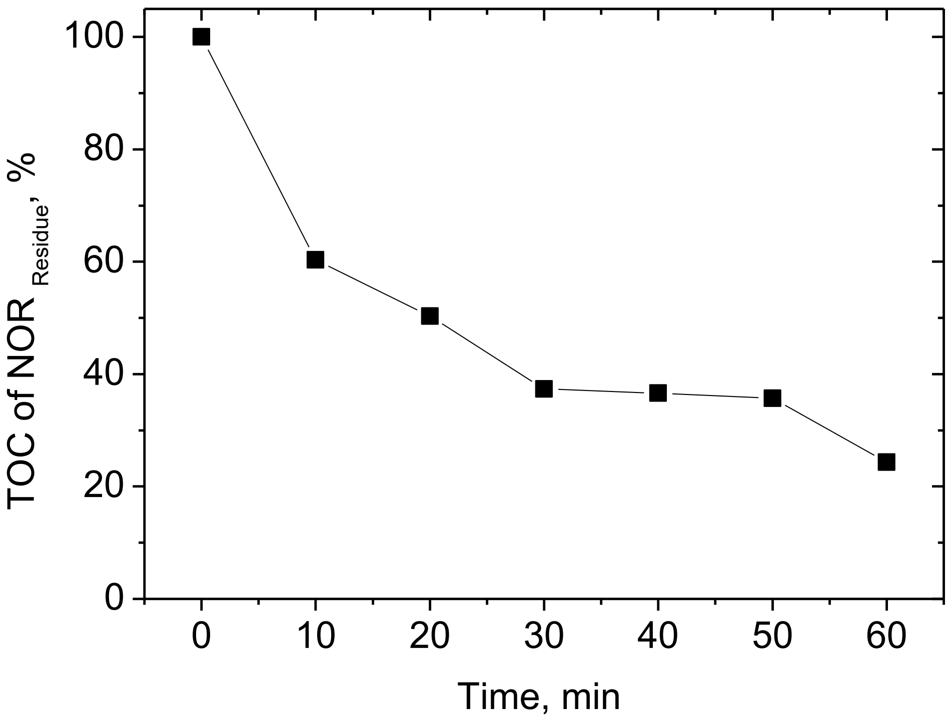

Figure SM-7. Removal of TOC during the degradation of NOR by the MW/UV process. (Experimental conditions: microwave power = 500 W, UV light intensity = 31.8 mW/cm^2, maximum wavelength = 254 nm, [NOR] = 5 mg/L, and pH = 6.72).
